# Drug-Induced Delirium Associated With Sacubitril/Valsartan in a Patient With Chronic Kidney Disease: A Case Report

**DOI:** 10.7759/cureus.72524

**Published:** 2024-10-28

**Authors:** Oscar Y Pena, Kim Kyung

**Affiliations:** 1 Medicine, Qunnipiac University Frank Netter School of Medicine, Bridgeport, USA; 2 Internal Medicine, St Vincent Medical Center, Bridgeport, USA

**Keywords:** general nephrology, general nephrology dialysis and transplanation, hfref: heart failure with reduced ejection fraction, sacubitril-valsartan, toxic delirium, visual hallucination

## Abstract

Drug-induced neuropsychiatric symptoms are not uncommon, particularly in patients with multiple comorbidities and polypharmacy. Sacubitril/valsartan is a combination medication used in the treatment of heart failure, especially for reducing hospitalizations in patients with heart failure with preserved ejection fraction (HFpEF). However, its neurological side effects, including delirium, hallucinations, and confusion, have been increasingly reported. This case report explores the occurrence of drug-induced delirium, specifically visual hallucinations, in a 79-year-old male with HFpEF and end-stage kidney disease (ESKD) after starting sacubitril/valsartan.

## Introduction

Drug-induced neuropsychiatric symptoms are not uncommon, particularly in patients with multiple comorbidities and polypharmacy. Sacubitril/valsartan is a combination medication used in the treatment of heart failure, especially for reducing hospitalizations in patients with heart failure with preserved ejection fraction (HFpEF). However, its neurological side effects, including delirium, hallucinations, and confusion, have been increasingly reported. This case report explores the occurrence of drug-induced delirium, specifically visual hallucinations, in a 79-year-old male with HFpEF and end-stage kidney disease (ESKD) after starting sacubitril/valsartan [1-3]. 

## Case presentation

A 79-year-old male with a history of chronic obstructive pulmonary disease (COPD), coronary artery disease (CAD), end-stage kidney disease (ESKD) on thrice-weekly hemodialysis, and heart failure with preserved ejection fraction (HFpEF) was admitted for acute hypoxic respiratory failure due to fluid overload. The patient was noted to have a significant right-sided pleural effusion and was treated accordingly. 

During hospitalization, the patient was initiated on sacubitril/valsartan for HFpEF management. On the fifth day of hospitalization, the patient developed acute confusion, confabulation, delusion, and visual hallucinations. These neuropsychiatric symptoms appeared within 24 hours of sacubitril/valsartan, initiation and decreased within 24 hours of its discontinuation. The patient had no prior history of psychiatric disorders or similar episodes.

A comprehensive evaluation of the cause of the delirium was undertaken. Infectious, metabolic, and neurologic causes were ruled out, with laboratory results showing no significant abnormalities aside from those related to the patient's ESKD. Brain imaging revealed no acute intracranial findings, and toxicology screens were negative. The temporal relationship between the onset of symptoms and the initiation of sacubitril/valsartan, combined with symptom resolution upon its discontinuation, strongly suggested drug-induced delirium.

Upon recognition of the temporal association between sacubitril/valsartan administration and the onset of visual hallucinations, the medication was promptly discontinued. No recurrence of neuropsychiatric symptoms was observed after cessation. The patient's other medications were reviewed, and no other drugs were implicated as a potential cause of the delirium.

The patient's delirium and hallucinations resolved entirely following the cessation of sacubitril/valsartan [1-3]. He remained stable for the remainder of his hospital stay, with no further neurological or psychiatric complications (Table 1). He was discharged with a modified medication regimen, excluding sacubitril/valsartan, and plans for close outpatient follow-up with both cardiology and nephrology [1-3].

**Table 1:** Sacubitril/Valsartan-Related Delirium and Symptom Resolution Timeline HFpEF: Heart Failure with perserved Ejection Fraction

Date	Event
Admission	Admitted for hypoxic respiratory failure due to fluid overload
Day 1	No psychiatric symptoms, no visual hallucinations
Day 4	Sacubitril/valsartan initiated for HFpEF
Day 5	Delirium and visual hallucinations developed
Day 6	Sacubitril/valsartan discontinued, symptoms resolved
Day 7	No recurrence of hallucinations post-discontinuation

## Discussion

This case underscores the potential for sacubitril/valsartan, to induce neuropsychiatric symptoms, such as visual hallucinations and delirium, in susceptible individuals [2]. The exact mechanism by which sacubitril/valsartan might contribute to these adverse effects remains unclear, but it is likely related to the patient’s complex comorbid conditions, including ESKD, which may alter drug metabolism and clearance [3].

Several reports in the literature have associated sacubitril/valsartan with psychiatric symptoms, including confusion, delirium, and hallucinations [2]. In patients with ESKD and other chronic conditions, medication management becomes more intricate due to altered pharmacokinetics, making them more prone to adverse drug reactions [3]. This case highlights the importance of thorough medication review and a high degree of clinical suspicion when managing unexpected neuropsychiatric symptoms [2].

## Conclusions

In patients with complex medical histories, including HFpEF and ESKD, medications such as sacubitril/valsartan, can lead to adverse neuropsychiatric effects. Prompt recognition and discontinuation of the offending agent can lead to rapid symptom resolution. This case highlights the need for vigilant monitoring of medication effects and judicious prescribing in patients with multiple comorbidities.
